# 脂质体转染UCH-L1 siRNA对肺腺癌细胞系H157细胞增殖和凋亡的影响

**DOI:** 10.3779/j.issn.1009-3419.2010.04.04

**Published:** 2010-04-20

**Authors:** 肖杰 屈, 衍富 王

**Affiliations:** 116011 大连，大连医科大学附属第一医院呼吸内科 Department of Respiratory Medicine, 1^st^ Hospital Affiliated to Dalian Medical University, Dalian 116011, China

**Keywords:** 泛素羧基末端水解酶-1, siRNA, 肺肿瘤, Ubiquitin C terminal hydrolase-L1, siRNA, Lung neoplasms

## Abstract

**背景与目的:**

泛素羧基末端水解酶-1（ubiquitin C terminal hydrolase-L1, *UCH-L1*）是近期研究较多的一类癌基因，在多种恶性肿瘤如食管癌、肺癌、乳腺癌等中均有异常表达和活性增强。本研究旨在探讨脂质体转染UCH-L1 siRNA对人肺腺癌H157细胞增殖、凋亡的影响。

**方法:**

人工合成抑制*UCH-L1*基因的siRNA片段，通过脂质体转染到H157细胞内，显微镜下观察其形态学变化；流式细胞术检测各组细胞周期变化和凋亡指数；RT-PCR检测UCH-L1 mRNA的表达；Western blot检测UCH-L1蛋白表达。

**结果:**

转染siRNA后的H157细胞，细胞增殖受到显著抑制，细胞出现明显凋亡，UCH-L1 mRNA表达显著下降，UCH-L1蛋白水平显著下降。

**结论:**

应用RNA干扰靶向抑制*UCH-L1*基因可以显著抑制H157细胞增殖，促进调亡。*UCH-L1*可能成为肺癌基因治疗的一个新靶点。

## Abstract (en)

**Background and objective:**

Several reports demonstrated that the ubiquitin C terminal hydrolase-L1 (*UCH-L1*) has been found to be an oncogene in malignant tumors such as esophageal carcinoma, lung cancer and breast cancer. The aim of this study is to explore the effects of liposomal transfection of UCH-L1 siRNA on the proliferation and apoptosis of lung adenocarcinoma cell lines H157.

**Methods:**

UCH-L1 siRNA was synthesized and transfected into H157 cell by liposome. Cell morphological change was observed with microscope, and cell proliferation and apoptosis index detected by flow cytometry, UCH-L1 mRNA expression was determined by RT-PCR and protein level of UCH-L1 was determined by Western blot.

**Results:**

For the H157 cell transfected with siRNA, cell proliferation was inhibited significantly, cell apoptosis appeared obviously, the expression of UCH-L1 mRNA and protein level of UCH-L1 significantly decreased.

**Conclusion:**

UCH-L1 siRNA is able to inhibit the proliferation of lung adenocarcinoma cell lines H157 and induce the apoptosis. *UCH-L1* might become a new target for lung carcinoma gene therapy.

泛素羧基末端水解酶-1（ubiquitin C terminal hydrolase-L1, UCH-L1），又名蛋白基因产物（PGP9.5），是泛素-蛋白酶体系中的重要成员，在细胞的蛋白水解途径中起调节细胞周期和死亡的重要作用。UCH-L1为泛素水解酶，它广泛表达于神经元分化的各个阶段，为神经内分泌系统的一个特定组织标志物。UCH-L1介导的针对细胞蛋白的泛素-蛋白酶体系统，可能是调节细胞周期基因的重要机制，通过引起细胞周期蛋白的去泛素化增加而促进未分化的体细胞无序生长和繁殖，同时UCH-L1的表达可能诱导一些能影响细胞分裂周期的基因^[1-6]^。因此，UCH-L1在调节细胞周期蛋白表达方面具有重要作用，从而与恶性肿瘤发生、发展及预后有重要关系。

Hibi等^[1]^应用SAGE法观察到UCH-L1在原发性肺癌和肺癌细胞株中高表达，但是在正常肺组织中未检测到。UCH-L1表达不仅与小细胞肺癌发生、进展及预后有关，而且也与非小细胞肺癌发生、进展及预后有密切关系。高表达者进展快、易发生转移、侵袭潜力强及预后差，可见UCH-L1的表达是不依赖神经分泌而独立存在的与肺癌病理分期及预后相关的重要生物学指标。为了探讨靶向UCH-L1治疗在肺癌发生发展中的意义和价值，评价siRNA介导的基因治疗技术，本实验设计合成特异性靶向治疗UCH-L1的siRNA分子，并将其转染至UCH-L1阳性表达肺癌细胞株H157细胞，观察其对UCH-L1表达的阻抑作用及其对肺癌细胞凋亡、增殖的影响。

## 1 材料与方法

### 1.1 主要实验材料

人肺腺癌H157细胞由大连医科大学病理科惠赠。UCH-L1 siRNA、UCH-L1及β-actin鼠抗人单克隆抗体购自Santa公司，脂质体（Lipofectamine^TM^ 2000）购自Invitrogen公司，IMDM培养液、胎牛血清购自GIBCO公司，UCH-L1上下游引物及RT-PCR相关试剂盒购自Takara公司。Trizol试剂、蛋白提取液、蛋白定量试剂盒及发光液ECL购自碧云天公司，辣根过氧化物酶（HRP）标记的羊抗鼠IgG购自北京中杉金桥公司。

### 1.2 细胞株的培养

人肺腺癌H157细胞在含10%胎牛血清、青霉素（100 μg/mL）、链霉素（100 μg/mL）的IMDM中培养，温度为37 ℃，CO_2_浓度为5%。

### 1.3 UCH-L1 siRNA分子的设计与合成

UCH-L1 siRNA分子由Santa公司设计并合成，该序列经BLAST查询，确定为UCH-L1特异性序列，排除与其它基因同源。阴性对照为由Santa公司提供的乱序序列。

### 1.4 细胞分组与转染

取对数生长期的H157细胞，传代接种于6 cm培养皿内，调整细胞浓度，使每孔细胞数为1×10^5^个，常规条件下培养24 h，在显微镜下观察，细胞融合达到80%时开始实验。设置：①空白对照组；②阴性对照组：转染阴性对照乱序siRNA序列；③siRNA转染组：转染特异性siRNA序列；④方法：按Invitrogen公司提供的转染试剂手册操作。

### 1.5 流式细胞术检测细胞周期及细胞凋亡

将收获的各组细胞制成单细胞悬液，加入预冷的冰乙醇，4 ℃内固定24 h后使用；将固定好的细胞，碘化丙啶溶液避光染色30 min后待用；采用流式细胞仪进行检测，以488 nm氩离子激光激发，采用Expo 32 ADC软件进行免疫荧光数据分析。

### 1.6 RT-PCR术检测UCH-L1 mRNA水平

收集培养细胞，调整细胞浓度为5×10^6^个/孔-1×10^7^个/孔，采用Trizol法提取总RNA。采用10 μL反应体系（含待测RNA 1 μL，AMV 0.5 μL及适当浓度的Oligod T、dNTP和氯化镁）30 ℃反应10 min，42 ℃反应45 min，99 ℃反应5 min，5 ℃反应5 min以合成cDNA。PCR反应体系采用40 μL反应体系（5×PCR Buffer 10 μL，Taq酶0.25 μL，引物各0.5 μL），PCR反应条件为94 ℃反应2 min，49.5 ℃反应45 s，72 ℃反应2 min，共经过35个循环反应。所用的UCH-L1的引物序列：上游为5'-CCCGAGATGCTGAACAAA-3'；下游为5'-GCCACTGCGTG AATAAGT-3'；产物为272 bp。同时以β-actin作为内参照，其引物序列为：上游：5'-GTGGGGCGCCCC AGGCACCA-3'，下游：5'-CTCCTTAATGTCACGC ACGATTTC-3'；产物为540 bp。扩增产物经含EB的1%琼脂糖凝胶电泳分离，紫外线下观察并应用Labworks软件分析计算各条带的积分光密度（integrate optical density, IOD）以UCH-L1与β-actin的IOD比值表示UCH-L1 mRNA的相对含量。

### 1.7 Western blot法检测UCH-L1蛋白水平

收集培养细胞，应用细胞蛋白提取液及PMSF（两者比例是1 000:1）提取蛋白，将蛋白定量后，转到PVDF膜，膜经5%脱脂奶粉封闭1 h后，分别加UCH-L1和β-actin鼠抗人抗体4 ℃孵育过夜，二抗室温孵育1 h，将PVDF膜进行化学发光，暗室中洗片机洗片。Labworks软件分析计算各条带的IOD，以UCH-L1与β-actin的IOD比值表示UCH-L1蛋白的相对含量。实验重复3次。

### 1.8 统计学处理

每组数据表示采用Mean±SD表示，采用SPSS 13.0软件进行单因素方差分析，*P* < 0.05为差异有统计学意义。

## 2 结果

### 2.1 细胞形态学观察

倒置显微镜观察转染前后细胞形态的变化：转染前，细胞贴壁较好，呈扁平多角形，中间有圆形的核，核内有一至多个核仁；转染后48 h，细胞体积收缩，贴壁不佳，细胞突起消失（见图 1）。

**1:**
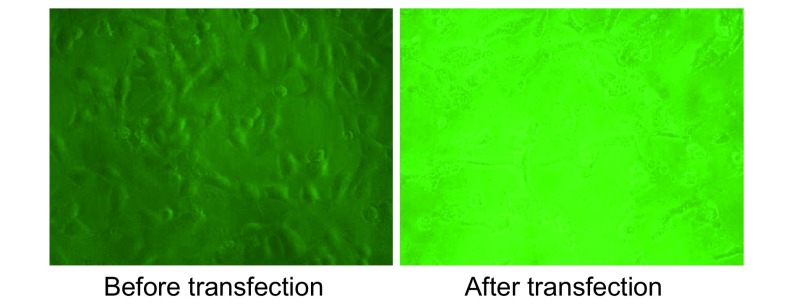
荧光显微镜显示转染前后细胞形态学变化 Cell morphological changes observed with microscope

### 2.2 流式细胞术检测细胞周期及细胞凋亡

于转染48 h后收集细胞检测细胞周期和凋亡，结果显示，UCH-L1 siRNA转染组细胞明显被阻滞于G_2_/M期，三组的凋亡率分别为UCH-L1 siRNA转染组（28.06±1.58）%、空白对照组（0.45±0.09）%、阴性对照组（0.52±0.07）%，空白对照组和阴性对照组间差异无统计学意义（*P* > 0.05），siRNA转染组与两个对照组间比较差异均有明显统计学意义（*P* < 0.001）。这说明UCH-L1 siRNA可以明显抑制UCH-L1，诱导细胞凋亡（图 2）。

**2:**
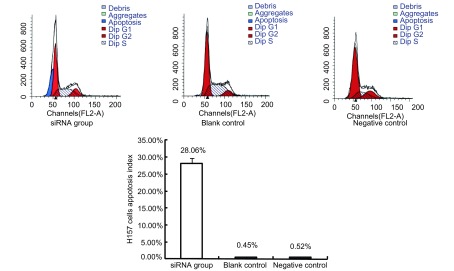
H157流式细胞术显示H157细胞凋亡情况 H157 cell apoptosis index detected by flow cytometry

### 2.3 UCH-L1 siRNA转染对H157细胞UCH-L1 mRNA的影响

通过RT-PCR可获得272 bp（UCH-L1）和540 bp（β-actin）两条带，β-actin在各组中表达相似、条带清晰，siRNA转染组UCH-L1表达的条带亮度低于其余各组，经Labworks软件分析系统鉴定电泳结果后，得出UCH-L1/β-actin吸光度比值：空白对照组分别为0.523±0.090；阴性对照组分别为0.520±0.108，这两个对照组间比较差异无统计学意义（*P* > 0.05）。siRNA转染组比值分别为0.260±0.037，与两个对照组比较差异均有统计学意义（*P*=0.009）。结果表明，UCH-L1 siRNA作用于H157细胞48 h后UCH-L1 mRNA表达明显下调，而空白对照组、阴性对照组UCH-L1 mRNA水平无明显变化。表明UCH-L1 siRNA可诱导UCH-L1 mRNA特异性降解（见图 3）。

**3:**
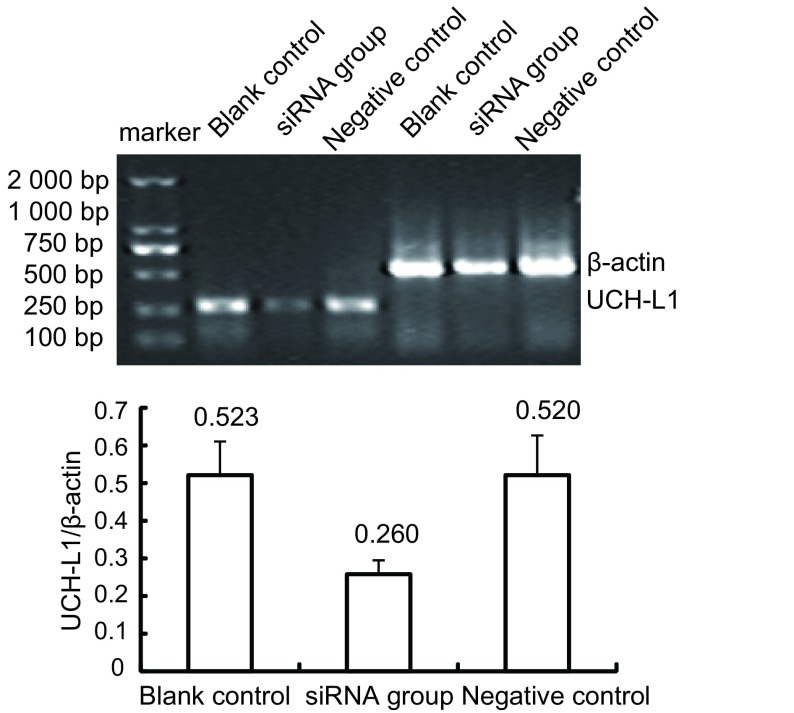
RT-PCR检测H157细胞UCH-L1的表达 Transcription expression of *UCH-L1* gene in H157 cells detected by RT-PCR

### 2.4 UCH-L1 siRNA转染对H157细胞UCH-L1蛋白水平的影响

通过Western blot可获得25 kDa（UCH-L1）和43 kDa（β-actin）两条带，β-actin在各组中表达相似、条带清晰，siRNA转染组UCH-L1表达的条带亮度低于其余各组，经Labworks软件分析系统鉴定结果后，得出UCH-L1/β-actin吸光度比值：空白对照组0.739±0.053；阴性对照组0.661±0.065，这两个实验组间比较差异无统计学意义（*P* > 0.05）。siRNA转染组比值为0.254±0.041，与两个对照组比较差异均有统计学意义（*P* < 0.001）。结果表明，UCH-L1 siRNA作用于H157细胞48 h后UCH-L1蛋白表达明显下调，而空白对照组、阴性对照组UCH-L1蛋白水平无明显变化。表明UCH-L1 siRNA可诱导UCH-L1蛋白特异性降解（图 4）。

**4:**
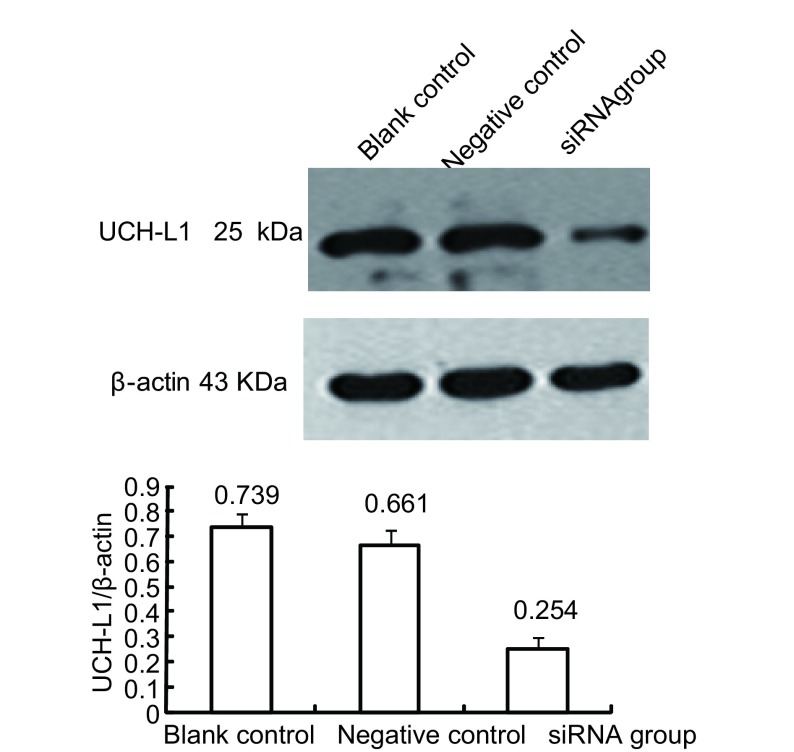
Western blot检测H157细胞UCH-L1的表达 UCH-L1protein expression detected in H157cell lines by Western blot

## 3 讨论

细胞凋亡对细胞生长和肿瘤发生起着重要的作用，许多研究表明凋亡调节功能的异常与肿瘤的发生有关，其中多个癌基因、抑癌基因在细胞凋亡途径中起作用。UCH-L1是由223个氨基酸组成的一类半胱氨酸水解酶，分子量约25 kDa。泛素-蛋白水解酶体途径^[7]^（ubiquitin proteasome pathway, UPP）是细胞质和核内蛋白ATP依赖性的非溶酶体降解途径，高效并高度选择性地进行细胞内蛋白转换，还参与某些重要蛋白质的编译后修饰和改造。因此，对维持细胞正常生理功能具有十分重要的意义。UCH-L1通过泛素-蛋白水解酶体途径调节细胞的增生、分化与凋亡，调节细胞周期蛋白的表达，从而与恶性肿瘤发生发展及预后有重要关系。

UCH-L1在多种恶性肿瘤中均有表达，Takase等^[6]^在40例切除的食管癌（鳞状细胞癌）中发现19例（48%）有UCH-L1的表达。Otsuki等^[8]^表明UCH-L1在骨髓瘤细胞中表达，并表明UCH-L1可能是血液系统恶性肿瘤中骨髓瘤的一个很好的标志物。Takano等^[5]^应用RT-PCR技术检测80例甲状腺髓样癌组织中UCH-L1 mRNA的表达水平，结果发现在11例遗传性和散发性甲状腺癌中出现UCH-L1 mRNA过度表达。Miyoshi等^[9]^通过研究乳腺癌组织中UCH-L1和UCH-L3的mRNA表达水平，表明在乳腺癌中UCH-L1和UCH-L3 mRNA高表达与预后不良有关。Tezel等^[10]^发现胰腺癌组织中UCH-L1的高度表达与术后生存期有着重要的反向关系。并表明UCH-L1可能成为胰腺癌患者手术预后的标志物。最近，Tanaka等^[11]^发现肾癌细胞高转移组织中UCH-L1的表达下调，提示UCH-L1的表达似乎与人类肾癌细胞SN12C的转移潜能有关。

UCH-L1在肺癌细胞中也有表达，Bittencourt等^[12]^表明在肺癌和肺癌细胞株中有UCH-L1的高表达。Sasaki等^[13]^应用RT-PCR研究95例非小细胞肺癌（non-small cell lung cancer, NSCLC）中UCH-L1的表达情况，结果表明有18例（12.8%）检测到UCH-L1转录，而正常肺组织中仅有一些微弱表达。而且UCH-L1表达与年龄、性别、身体状况或者病理类型无关，但是相对于T1/T2 NSCLC（6/54, 11.1%），UCH-L1优先表达于T3/T4 NSCLC（12/41, 29.3%）。这说明UCH-L1可能与NSCLC的发展和侵袭有关。这些结果为癌症的选择性治疗提供了一个新的途径。

RNA干扰^[14]^（RNA interference, RNAi）是近年来发展起来的新技术，可高效、特异地抑制基因的表达，是高度特异的mRNA水平上的转录后基因沉默机制，成为后基因组时代功能基因组学研究中不可缺少的重要手段，在探查基因功能、探索肿瘤的发生、发展机制、感染性疾病和显性基因病等方面有广泛的应用前景，成为近年来分子生物学领域的研究热点。

目前，只有极少数研究应用RNA干扰技术敲除恶性肿瘤UCH-L1。Wang等^[15]^为了澄清人类肿瘤细胞中UCH-L1的作用，分别用pcDNA 3.1-UCH-L1质粒和UCH-L1 siRNA转染人乳腺癌MCF7和MCF7/Adr细胞系，实验表明由UCH-L1引起的细胞凋亡过程在某种程度上可能是通过PI3K/Akt信号通道。Rolén等^[16]^应用RNAi技术敲除UCH-L1来抑制BL细胞的增殖，实验表明UCH-L1的酶活性作用参与了信号传导途径并促进恶性B细胞的增殖和侵袭能力。Anjali等^[17]^通过UCH-L1 siRNA干扰被EB病毒感染的B细胞和被肉瘤病毒40感染的293型人胚肾细胞从而证实UCH-L1参与了肿瘤的增殖与侵袭，同时抑制细胞凋亡。

而且，应用RNA干扰技术研究肺癌细胞中UCH-L1的表达以及与肺癌发生机制的关系只有2例。Kim等^[18]^经实验发现UCH-L1在NSCLC H157细胞株内高表达，并具有高侵袭力，同时也发现肿瘤细胞内UCH-L1的表达促进了肿瘤细胞体内外的侵袭能力，UCH-L1通过Akt介导通道调节细胞粘附从而改变了细胞结构。应用RNAi技术抑制UCH-L1表达就抑制了肿瘤细胞在体内外的侵袭，也抑制了Akt的活性。而如果Akt突变，则UCH-L1高表达不会影响H157细胞的侵袭和转移能力。这就说明UCH-L1是一关键分子，并且依靠上调Akt活性来调节肿瘤细胞的侵袭能力。Liu等^[19]^研究表明，应用RNAi技术抑制H1299肺肿瘤细胞株UCH-L1的表达，会促进肿瘤细胞增殖，同时表明UCH-L1的表达是预防增殖的、针对肿瘤生长的一种反应，而这一反应的分子生物学机制有待于进一步研究。

为了进一步探索应用特异性siRNA干扰肺癌细胞UCH-L1后肺癌的发生、发展及转移情况，本研究成功构建了针对UCH-L1的siRNA，并通过脂质体转染UCH-L1表达阳性的H157细胞，观测其对细胞UCH-L1表达及细胞增殖、凋亡的影响。流式细胞术实验结果证实UCH-L1 siRNA转染可有效抑制人肺腺癌H157细胞的增殖活性，阻滞H157细胞于G_2_/M期，诱导细胞凋亡。RT-PCR结果显示，UCH-L1 siRNA转染可显著抑制UCH-L1 mRNA的表达。Western blot结果证明UCH-L1 siRNA转染H157细胞后可以有效抑制UCH-L1蛋白表达，表明UCH-L1参与了H157细胞周期和增殖调控，在NSCLC的发展中具有重要作用。

虽然本研究证实了应用RNA干扰技术抑制UCH-L1表达的可行性，研究结果为应用siRNA进行肺腺癌基因治疗的进一步研究提供了实验基础和方向，然而，应用脂质体瞬时转染存在脂质体毒性较大、转染效率低、基因沉默效应短暂等不足，还需要构建带筛选标记的siRNA表达质粒，进行稳定转染，以获得较长时间的基因沉默效应和更为理想的干扰效果。
